# Regulation of the PI3K/Akt pathway during decidualization of endometrial stromal cells

**DOI:** 10.1371/journal.pone.0177387

**Published:** 2017-05-05

**Authors:** François Fabi, Kathy Grenier, Sophie Parent, Pascal Adam, Laurence Tardif, Valérie Leblanc, Eric Asselin

**Affiliations:** Department of Medical Biology, Université du Québec à Trois-Rivières, Trois-Rivières, Québec, Canada; South China Agricultural University, CHINA

## Abstract

Infertility is constantly increasing in Canada, where 16% of Canadian couples are experiencing difficulty conceiving. It is thought that infertility can emanate from the dysregulated communication between the embryo and the maternal endometrium. In order to allow for this window of implantation to be open at the right moment, endometrial stromal cells proliferate and differentiate by a mechanism called decidualization. Intracellular and molecular mechanisms involved in the regulation of apoptosis and cell proliferation during decidualization of the endometrium are yet to be fully understood. It has been well demonstrated previously that Akt is importantly involved in cell survival and glycogen synthesis. Akt1, Akt2 and Akt3 isoforms have distinct physiological roles; this could also be the case during decidualization and pregnancy. The aim of this study is to investigate the regulation of PI3K/Akt pathway during the decidualization process of endometrial stromal cells. Expression of Akt isoforms, Akt activity (phospho-Akt), pIκB and substrates of Akt during decidualization were measured. To our knowledge, these results are the first to suggest a decrease in levels of Akt isoforms as well as a downregulation of Akt activity in the process of decidualization of human endometrial stromal cells. We also uncovered that decidualization induced nuclear localization of p65 through the phosphorylation of IκB, its inhibitory subunit; however, Par-4, a recently uncovered regulator of cell differentiation, was displaced from the nucleus upon decidualization. Our results also suggest that HIESC cells exhibit decreased motility during decidualization and that PI3K pathway inhibition could be involved in this process. Finally, we demonstrate that specific Akt isoforms present unique effects on the successful induction of decidualization. Further analyses will involve investigations to understand the precise signaling mechanisms by which this pathway is regulated.

## Introduction

Infertility is a problem that increasingly afflicts Canadian; in 2012, 16% of Canadian couples were found to have difficulties conceiving, a number that has doubled in the last 30 years[1]. The main cause of infertility can be traced to communication failure between the embryo and the maternal endometrium. This complex tissue constitutes the inner lining of the uterus and undergoes cyclic, subtle and intricate changes. During the late secretory phase of the menstrual cycle, endometrial stromal cells proliferate and differentiate by undergoing decidualization, a fundamental mechanism responsible for major changes in those cell phenotypes; morphological transformations occur to the fibroblast-like endometrial stromal cells that differentiate into polygonal, epithelial-like cells, becoming enlarged with lipids and glycogen secretions [2, 3]. This process of cellular differentiation is characteristic of mesenchymal to epithelial transition (MET) [4] and is confirmable by the decreased expression of mesenchymal markers such as Slug, Snail or Vimentin[5]. Various studies have shown that decidualization-induced change in phenotype exhibits many molecular characteristics of MET; while still insufficient, many evidences point to that process as a pivotal event in the stromal cell preparedness for embryo implantation[6–8]. Decidualization is a transitory phase of the endometrium that allows the decidua to feed and protect an early implanted embryo while waiting for a complete and functional placenta. It also allows the endometrium to become receptive to embryonic signaling that precedes and favorize implantation [9].

During each reproductive cycle, the decidualization process prepares the endometrium for the incoming embryo and, possibly, implantation. Further details can be found about decidualization and implantation processes in the following review [10]. Decidualization of stromal cells is induced through the concerted effect of cAMP and progesterone, or their respective analogs, 8-bromo-cyclic adenosine monophosphate (8-br-cAMP) and medroxyprogesterone acetate (MPA) [2, 11, 12]. Progesterone is necessary to induce as well as maintain the morphological and biochemical characteristics of the decidualization in a long term endometrial cell culture [11]; on the other hand, many studies have shown that cAMP main effect is to sensitize the cells to the action of the progesterone [13]. The combination of those two analogs induces faster morphological and biochemical changes than progesterone alone. All those changes are associated with an increased secretion of many proteins, some of which are only secreted in response to the differentiation of the endometrium; they are thus known as marker of decidualization [14, 15]. Two well-known successful decidualization markers are prolactin (PRL) and insulin growth factor binding protein-1 (IGFBP1), the secretion of the former being maximal when the cells are treated with the combination of 8-br-cAMP and MPA.

It has been previously demonstrated that Akt is involved in the cell survival of the rat endometrium [16, 17]. Akt, also known as protein kinase B (PKB), is a cytosolic serine/threonine kinase that promotes cellular survival and acts as a regulator of numerous cellular functions such as cell proliferation, growth, metabolism, angiogenesis and malignant transformation [18, 19]. Up to date, three isoforms of Akt have been identified and possibly have distinct roles during the different phases of pregnancy [20]. The three isoforms of Akt, Akt1/PKBα, Akt2/PKBβ and Akt3/PKBγ play different roles as shown by diverse experimentation with deficient mice involving each isoform. Each isoform is produced by distinct gene but exhibit an overall amino acids homology of 80%. [18]. All three phenotypes of knockout Akt1-2 or 3 mice are viable but the deletion of each Akt isoforms induces distinct metabolic and physiological changes. Mice devoid of Akt1 exhibit decreased cell survival observable by growth retardation and decreased in overall organ size and increased perinatal mortality [21, 22]; disruption of Akt2 causes reduction in insulin-stimulated glucose uptake in muscle and fat, so those mice are insulin intolerant and show diabetes-like symptoms [23]; on the other hand Akt3-deficient mice don't display these symptoms but present smaller brain size with a reduced total number of cell as well as decreased average cell size [24]. Although they possess differential as well as redundant effects, all three Akt isoforms are activated by a phosphatidylinositol 3-kinase (PI3K) in response to growth factors [25]. PI3-K is a heterodimers composed of two subunits, the catalytic subunit (p110) and an adaptor/regulatory subunit (p85). This kinase converts the phosphatidylinositol-3,4-diphosphate (PIP2) in phosphatidylinositol-3,4,5-triphosphate (PIP3), which interacts with the pleckstrin homology (PH) domain of the 3’-phosphoinositide-dependent kinase-1 (PDK1). This serine/threonine kinase is able to phosphorylate a threonine residue on Akt, who also binds to the PIP3 by its PH domain. Recruitment of Akt to the plasma membrane allows its full activation through phosphorylation of two crucial sites. A threonine residue (T308 on Akt1, T309 on Akt2 and T305 on Akt3) is phosphorylated in the catalytic domain by PDK1, and a serine residue (S473 on Akt1, S474 on Akt2 and S472 on Akt3) is phosphorylated in the C-terminus domain by the complex mTORC2 [26]. Upon full activation, Akt acts as a potent inducer of cell survival and proliferation which are both crucial to the fine regulation necessary for successful implantation.

The intracellular and molecular mechanisms implicated in the regulation of apoptosis and cell proliferation during decidualization of the endometrium are yet to be fully elucidated. Akt is known to regulate the cell survival but the contribution of each distinct isoform is unclear. Considering the current knowledge of these mechanisms, the purpose of this study is to determine the role of each isoform in the decidualization of the endometrium. Our results suggest that during decidualization, the protein level of each Akt isoform decreases while we also observed a decrease of phosphorylation and thus activation which culminated in the decrease of Slug, an EMT marker, and a decrease of the phosphorylation of mTOR, a known substrate of Akt. We also uncovered that decidualization induced the activation of the NF-κB pathway and allowed nuclear exclusion of Par-4, a known mediator of EMT. Using plasmids expressing constitutively active Akt during the decidualization process, we also established that Akt 1, 2 and 3 all delayed the production of decidualization markers PRL and IGFBP1. These results suggest that decidualization inhibits PI3K/Akt signaling pathway and activates the NF-κB pathway; these changes might induces partial, MET-like molecular changes. Expression of Akt may interfere in the decidualization process and could be a novel therapeutic target in the treatment of infertility.

## Materials and methods

### Cell culture

Human immortalized endometrial stromal cells (HIESC) were a kind gift from Michel A. Fortier (Centre de Recherche du Centre Hospitalier Université Laval (CHUL), Qc, Canada). Cells were cultured in RPMI medium (Thermo Scientific, Rockford, IL) supplemented with 10% FBS (Fetal Bovine Serum) and 50μg/ml of gentamycin. Cells were maintained at 37°C in an atmosphere of 5% CO_2_. Cells were maintained between 20% and 90% confluency at all times, including upon seeding.

### Reagents and antibodies

Proteasomal inhibitor (Mg132), Medroxyprogesterone 17-acetate (MPA), phosphatidylinositol 3-kinase (PI3K) inhibitor (Wortmannin) and mammalian target of Rapamycin (mTOR) inhibitor (Rapamycin) were obtained from Sigma-Aldrich (St. Louis, MO). Fetal bovine serum (FBS) and gentamycin sulfate were purchased from HyClone (South Logan, Utah). 8-Bromo-cAMP was obtained from Enzo Life Sciences Inc (Farmingdale, NY). Akt 1 (2938), Akt 2 (2964), AKT total (9272), phospho-AKT (4060), phospho-IκBα (2859), IκBα (9242), phospho-mTOR (2974), mTOR (2983), Par-4 (2328), PARP (9532), FoxO1 (2880), Slug (9585) and Ubiquitin (3933) primary antibodies were purchased from Cell Signaling (Danvers, MA). Anti-Akt 3 (07–383) was obtained from Millipore (Billerica, MA), beta-tubulin antibody and HRP-conjugated GAPDH(ab9484) antibodies were from Abcam (Cambridge, MA) and monoclonal anti-β-actin-peroxidase from Sigma-Aldrich (St. Louis, MO). Horseradish peroxidase (HRP)-conjugated anti-rabbit and anti-mouse secondary antibodies were provided by Bio-Rad Laboratories (Mississauga, ON, Canada). A full description of antibodies, catalog numbers and concentration can be found in S1 Table.

### *In vitro* decidualization induction of HIESC

HIESC cells were cultured in maintenance medium and then plated 5x10^5^ cells per wells. Confluent monolayers were treated in phenol red-free RPMI 1640 (Gibco^**®**^ by Life Technologies™) containing 2% dextran coated charcoal-treated FBS (DCC-FBS) and 50μg/ml of gentamycin with or without 0.5 mM 8-bromo-cAMP and 1 μM MPA to induce decidualization. The culture medium was changed every two days and cells were grown at 37°C under a humidified atmosphere of 5% CO2, supernatants were collected and cells were harvested at days 1, 3, 6 and 9.

### Prolactin assays

To confirm decidualization, prolactin levels in supernatant were measured at days 1, 3, 6 and 9 of decidualization. PRL assay measurements were conducted by EIA Kit (Cayman, Ann Arbor, MI) and the optical density was read withw Fluostar OPTIMA BMG spectrophotometer (BMG Labtech Inc.; Durham, NC) at 450 nm.

### Proteasomal inhibition

Proteasomal inhibition is used to determine if protein degradation occurs via ubiquitin-proteasome pathway. HIESC cells were seeded in six-wells plate and decidualization was induced in phenol red-free RPMI containing 2% dextran coated charcoal-treated FBS (DCC-FBS) and 50μg/ml of gentamycin with 0.5 mM 8-bromo-cAMP and 1 μM MPA. After 48 h, Mg132 was added in fresh decidualization medium and cells were incubated for another 24 h and then harvested.

### Protein extraction and western blot analysis

After each treatment HIESC cells were lysed in RIPA buffer (pH 7.4, 150 mM NaCl, 0.1% SDS, 0.5% sodium deoxycholate, 1% NP-40 in PBS) containing protease and phosphatase inhibitors (Complete™ and PhosSTOP from Roche Applied Science) and frozen-thawed three times, then centrifuged (13000 x g, 20 min at 4°C) to remove insoluble material. The supernatant was recovered and store at -20°C pending analysis. Equal amounts of protein extract (15 μg), as determined by Bio-Rad DC protein assay were loaded in each well and then resolved by SDS-PAGE. After that, proteins were transferred using a semi-dry cell onto nitrocellulose membranes (Bio-Rad, Hercules, CA). Membranes were blocked with 5% milk in PBS containing 0.05% Tween 20 for 1h at room temperature, probed overnight at 4°C with primary antibody, washed three times in PBS-0.05% Tween 20 and incubated with horseradish peroxidase-conjugated secondary antibody (Bio-Rad, Hercules, CA) for 45 minutes at room temperature. Hybridized membranes were washed three times in PBS-0.05% Tween 20 and protein detection was performed by detecting peroxidase activity using SuperSignal West Femto™ substrate (Thermo Scientific, Rockford, IL), as described by the manufacturer’s instructions. Signal was visualized using the BioImaging System (UVP, CA, USA). Nuclear/cytoplasmic fraction experiments followed identical protocols; however, lysis was instead performed using the NE-PER Nuclear and Cytoplasmic extraction kit (Thermo Scientific, Rockford, IL).

### Reverse transcription polymerase chain reaction (RT-PCR and qRT-PCR)

To measure the transcripts levels, total RNA was isolated from cells using RNeasy Mini Kit from QIAGEN (Mississauga, ON, Canada). Total RNA (1μg) was subjected to reverse transcription using qScript cDNA Supermix (Quanta Biosciences, Gaithersburg, MD) as described by the manufacturer’s instructions. The reverse-transcribed RNA was then amplified by PCR using specific primers. The expression of Akt isoforms (Akt 1, 2 and 3), prolactin and IGFBP1 (Insulin-like growth factor binding protein 1) mRNAs was measured. The primer pairs used were 5’-TCTATGGCGCTGAGATTGTG-3’ (forward) and 5’-CTTAATGTGCCCGTCCTTGT-3’ (reverse) for Akt 1, 5’-TGAAAACCTTCTGTGGGACC-3’ (forward) and 5’-TGGTCCTGGTTGTAGAAGGG-3’ (reverse) for Akt 2, 5’-TGAAGTGGCACACACTCTAACT-3’ (forward) and 5’-CCGCTCTCTCGACAAATGGA-3’ (reverse) for Akt 3, 5’-AAAGGATCGCCATGGAAAG-3’ (forward) and 5’GCACAGGAGCAGGTTTGAC-3’ (reverse) for prolactin and finally 5’-TTTTACCTGCCAAACTGCAACA-3’ (forward) and 5’-CCCATTCCAAGGGTAGACGC-3’ (reverse) for IGFBP1. Human β-actin was used as an internal control and the primers used were 5’-CCTCCCTGGAGAAGAGCTA-3’ (forward) and 5’-ACGTCACACTTCATGATGGA-3’ (reverse). Each reaction mixture (final volume, 25 μL) contained OneTaq^®^ Quick-Load^®^ 2X Master Mix with Standard buffer (12.5 μL) from NEW ENGLAND BioLabs, (Whitby, ON, Canada), 10 μM forward primer (0.5 μL), 10 μM reverse primer (0.5 μL), cDNA (2 μL) and nuclease-free water (9.5 μL). PCRs were performed in C1000 Touch™ Thermal cycler, a Bio-Rad system, following these specific parameters: 30 s at 95°C, 30 s at 60°C, 30 s at 68°C for 24 cycles (β-actin), 32 cycles for Akt 1, Akt 2 and Akt 3 and 33 cycles for IGFBP1 and prolactin. The PCR products obtained were separated through elecrophoresis on a 1% agarose gel, stained with SYBR-Safe (Invitrogen, Carlsbad, CA) and visualized with Gel Doc system (Bio-Rad). Real time PCR analyses were conducted using SensiFAST SYBR® Lo-ROX Kit (Bioline Reagents, MA) using an Mx3000P system (Agilent Technologies, Mississauga, Ontario, Canada). For each gene target, a standard curve was generated to determine the efficiency of the reaction, and the Pfaffl analysis method was used to measure the relative quantity of gene expression. Each real time PCR was performed in duplicates from at least three independent experiments. The above-described human prolactin, Akt1, Akt2, Akt3 primers were used; however, a sense primer 5’-TTGGGACGCCATCAGTACCTA-3’ and antisense primer 5’-TTGGCTAAACTCTCTACGACTCT-3’was used for IGFBP1. Either 18S with primer 5’-TGGTCGCTCGCTCCTCTCCC-3’ (forward) and 5’-CAGCGCCCGYCGGCATGTAT-3’ (reverse) or human β-actin were used as a reference gene based on their stable expression in all cell clones as an internal control. The Pfaffl method of quantification was used to measure relative expression.

### Production of HIESC cell lines with Tet-inducible constitutive active Akt isoforms

All three myristoylated Akt isoforms were cloned separately into pLVX-Tet3G-puro vector (Clontech) by InFusion Cloning (NEB). Briefly, first each Myr-Akt was PCR-amplified using specific primers with sequence homologous to each side of the construct. For sequence template for Myr-Akt, constitutively active (CA) Akt1 vector (pcDNA3-Myr-Akt1) was generously provided by Dr. Zhenguo Wu (Hong Kong University of Science and Technology). For Myr-Akt2 sequence template CA-Akt2 vector (pcDNA3-Myr-Akt2) was generously provided by Dr. Joseph R. Testa (Fox Chase Cancer Center, Philadelphia, PA). For Myr-Akt3, we used the CA-Akt3 vector (pcDNA3-Myr-Akt3) constructed in our laboratory [27] as template Each PCR fragment was inserted in the pLVX-Tet3G-puro vector linearized by restriction digestion and cloned as InFusion manufacturer’s instruction (Clontech). Vector were amplified in NEB Stable E.Coli and purified by MidiPrep (QIAGEN). These 3 new vectors and the pLVX-EF1a-Tet3G, were used to produce lentivirus with the Lenti-X 293T cell line and Lenti-X™ Lentiviral Expression Systems (Clontech). HIESC cells were cultured in maintenance medium and then plated 3x10^5^ cells per wells. Media was removed and replaced by media containing a combination of EF1a-Tet3G lentivirus and either Myr-Akt1, Myr-Akt2 or Myr-Akt3 lentivirus in the presence of 5.4 ug/ml retronectin, and incubated for 24 hrs at 37oC in 5%CO2. Then, media was refreshed and 24hrs of recovery later, a pool of transduced cells were selected in the presence of 100ug/ml G418 and 0.05ug/ml puromycin. Doxycycline-induction of Myr-Akt1, 2 and 3 isoforms in these cells in culture was confirmed by western blots using Akt1, 2 or 3 antibody and p-Akt(Ser473) antibody.

### In vitro decidualization of Tet-inducible HIESC cell lines

HIESC-Myr-Akt1, HIESC-Myr-Akt2 or HIESC-Myr-Akt3 cells were cultured in maintenance medium and then plated 5x10^5^ cells per wells. Confluent monolayers were treated in phenol red-free RPMI 1640 (gibco^**®**^ by life technologies™) containing 2% dextran coated charcoal-treated FBS (DCC-FBS) and 50μg/ml of gentamycin with or without 0.5 mM 8-bromo-cAMP and 1 μM MPA to induce decidualization. For Tet3G induction of Myr-Akt isoforms, 1ug/ml doxycycline was added to the culture media in appropriate wells. The culture medium was changed every two days and cells were grown at 37°C under a humidified atmosphere of 5% CO2, RNA from cells was harvested at days 3 and used for qPCR analysis of decidualization markers.

### Wound healing assay

Cells were seeded into six-well plates and allowed to grow in maintenance medium until they reached 90% confluence. Cells monolayers were scratched with the blunt end of a standard p10 pipette tip and dislodged cells were washed away with PBS. Cells were then incubated in phenol red-free RPMI containing 2% dextran coated charcoal-treated FBS (DCC-FBS), 50 μg/ml of gentamycin and one of the following: for decidualization induction: 0,5 mM 8-bromo-cAMP and 1 μM MPA, for PI3K inhibition: 1 μM Wortmannin, for mTOR inhibition: 100 nM Rapamycin. Migration was captured using an Olympus microscope at 0, 6, 12, and 24h post-wounding. Wound closure was quantified as the percentage of recovered area using Image J software.

### Immunofluorescence

Cells were seeded in 6-wells containing glass coverslips. After treatment, cell were fixed for 10 minutes in 4% PFA and then washed in PBS (pH7.2) for 10 minutes thrice. Cells were then permeabilised with 0.1% Triton X-100 PBS for 10 minutes and then washed in pH7.2 PBS for 10 minutes thrice. Cells were incubated overnight, in dark conditions, at 4°C with either p65 primary antibody (8242) (Cell Signaling, Danvers, MA), Par-4 primary antibody (HPA012640) (Sigma-Aldrich, St-Louis, MO) or the control Rabbit IgG (I-1000) (Vector Labs, Burlingame, CA) at the relevant concentration. Cells were then washed thrice in PBS and incubated at room temperature for 90 minutes in presence of Alexa Fluor 555 anti-rabbit antibodies (A21428) (Thermo Scientific, Rockford, IL). Cells were washed once using PBS and then counterstained with Alexa Fluor 488 Phalloidin (A12379) for 20 minutes at room temperature in dark conditions in order to mark F-actin; cells were again washed once using PBS and finally counterstained with Hoechst 33248 (1:10000 dilution) for 5 min and washed twice with PBS. Slides were then mounted using Slowfade gold antifading reagent (Thermo Scientific, Rockford, IL) and viewed using a TCS SP8 Leica confocal microscope at 63x. A full description of antibodies, catalog numbers and concentration can be found in S1 Table.

### Statistical analysis

All the experiments were repeated three times. Densitometric analyses were performed using Quantity One software (Bio-Rad). All data were either subjected to one-way ANOVA (PRISM software version 5.0; GraphPad, San Diego, CA) followed by Tukey's test or Student’s t-test to determine the differences between the experimental groups. Differences were considered significant at the level of p < 0.05.

## Results

### Decidualization of HIESC

**Fig 1:**
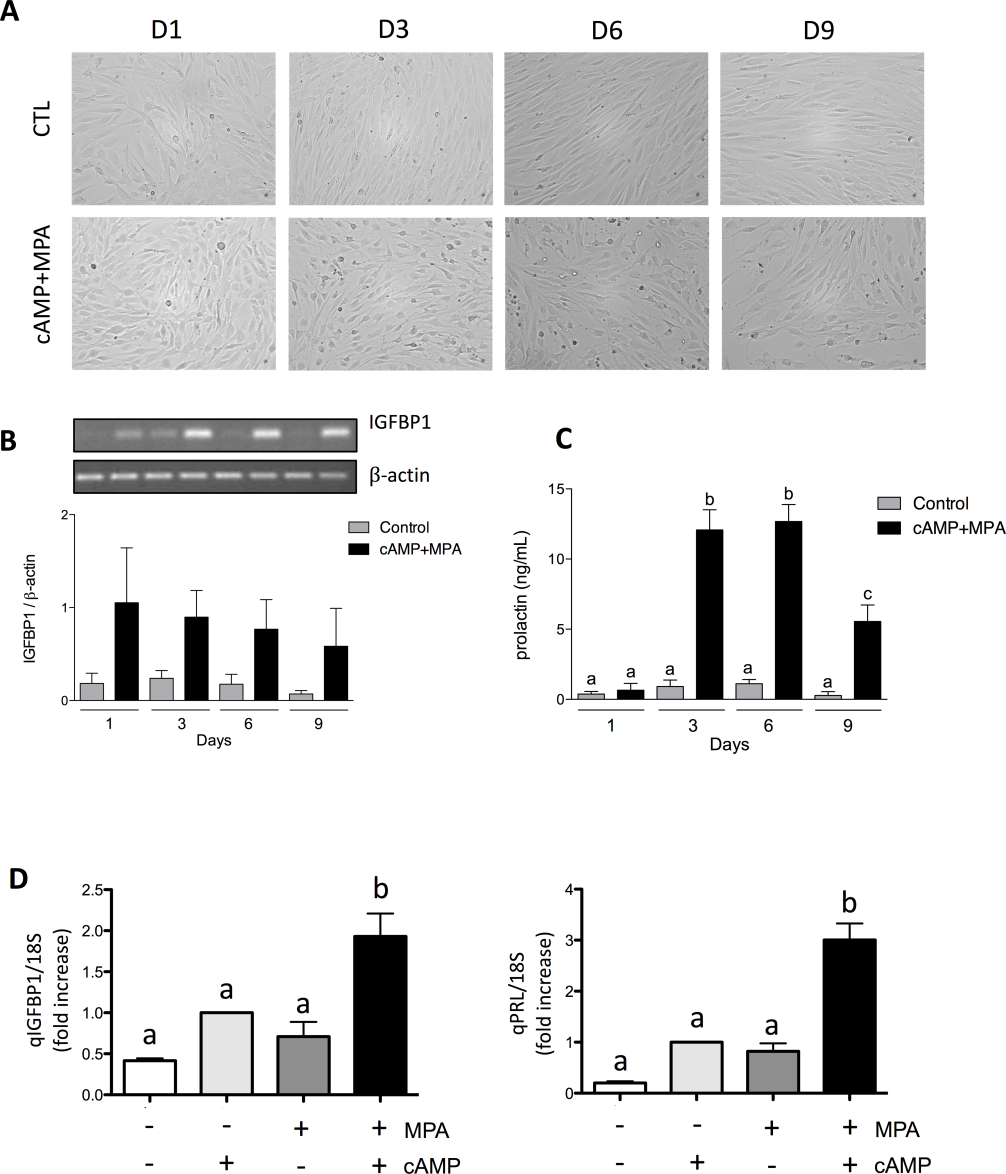
Induction of decidualization. (A) Treatment with cAMP (0.5 mM) and MPA (10^-6^M) for 9 days induced a morphological change in HIESC from a spindle to an ovoid shape. Images were taken using an Olympus BX60 microscope at 40x magnification. (B) mRNA were analysed by RT-PCR. β-actin was used as an internal control. Image shown are from one representative experiment. Graphics represent densitometric analysis. (C) Induction of PRL secretion by the same treatment in HIESC at different days of culture (1, 3, 6 and 9). A significant increase was found at days 6 and 10 with maximal levels observed at day 6 (P<0.0001). Expression of decidual marker genes, IGFBP1 (D) mRNA expression of either PRL or IGFBP1 were quantified following three day treatments. Cells were incubated in the presence of either cAMP (0.5 mM), MPA (10μM) or a combination of both. Cells were lysed after three days, RNA was extracted and qRT-PCR analyses were performed to quantify PRL or IGBP1 expression. 18S mRNA expression was used as control for qPCR results. All data are means ± SEM three independent experiments. Different letters represent significantly different means (*p*<0.05).

MPA and cAMP are known inducer of decidualization in endometrial stromal cells, a process upon which the stromal cell adopt an epithelioid phenotype. To confirm that the decidualization process occurred using cAMP and MPA treatment in HIESC cells, we analyzed cell morphology and measured the production of known well-characterized decidual markers (IGFBP1 and PRL). Upon concomitant treatment of cAMP and MPA, we observed a clear morphological changes characteristic of decidual cells (Fig 1A). Cells became enlarged, somewhat polygonal, and full of lipids and glycogen. The usually spindle-shaped stromal cells differentiated into ovoid cells displaying abundant cytoplasm; the sum of these changes can be described as a transition from mesenchymal to epithelial shape. We also measured mRNA expression of the decidual gene marker IGFBP1. Densitometric analyses of qRT-PCR analysis revealed a significant increased expression of IGFBP1 (Fig 1B) mRNA following cAMP+MPA treatments; on the other hand, the decidualization treatment significantly stimulated the secretion of PRL, a decidual marker of crucial importance, to its maximum on day 6 (12,69 ± 2.07 ng/mL) then PRL decreased to reach 5,55 ± 2,04 ng/mL on day 9 (Fig 1C). Finally, in order to ascertain that the increased expression of PRL and IGFBP1 mRNA was truly caused by the concomitant use of both cAMP and MPA and not their singular use, further RT-qPCR experiments were conducted. The effect of single administration of cAMP (0.5mM), MPA (1μM) and their combination was compared and mRNA expression of IGFBP1 and PRL was measured (Fig 1D); both markers were shown to be significantly increased only in the context of the combined use of cAMP and MPA. Together, these results strongly suggest that the combined use of cAMP and MPA is effective and indispensable in inducing decidualization in HIESC cells. The obtained results also suggested that day 3 of decidualization was optimal for further experiments, considering that prolactin secretion and IGFPB1 expression were optimal.

### Expression of Akt isoforms during *in vitro* decidualization

**Fig 2:**
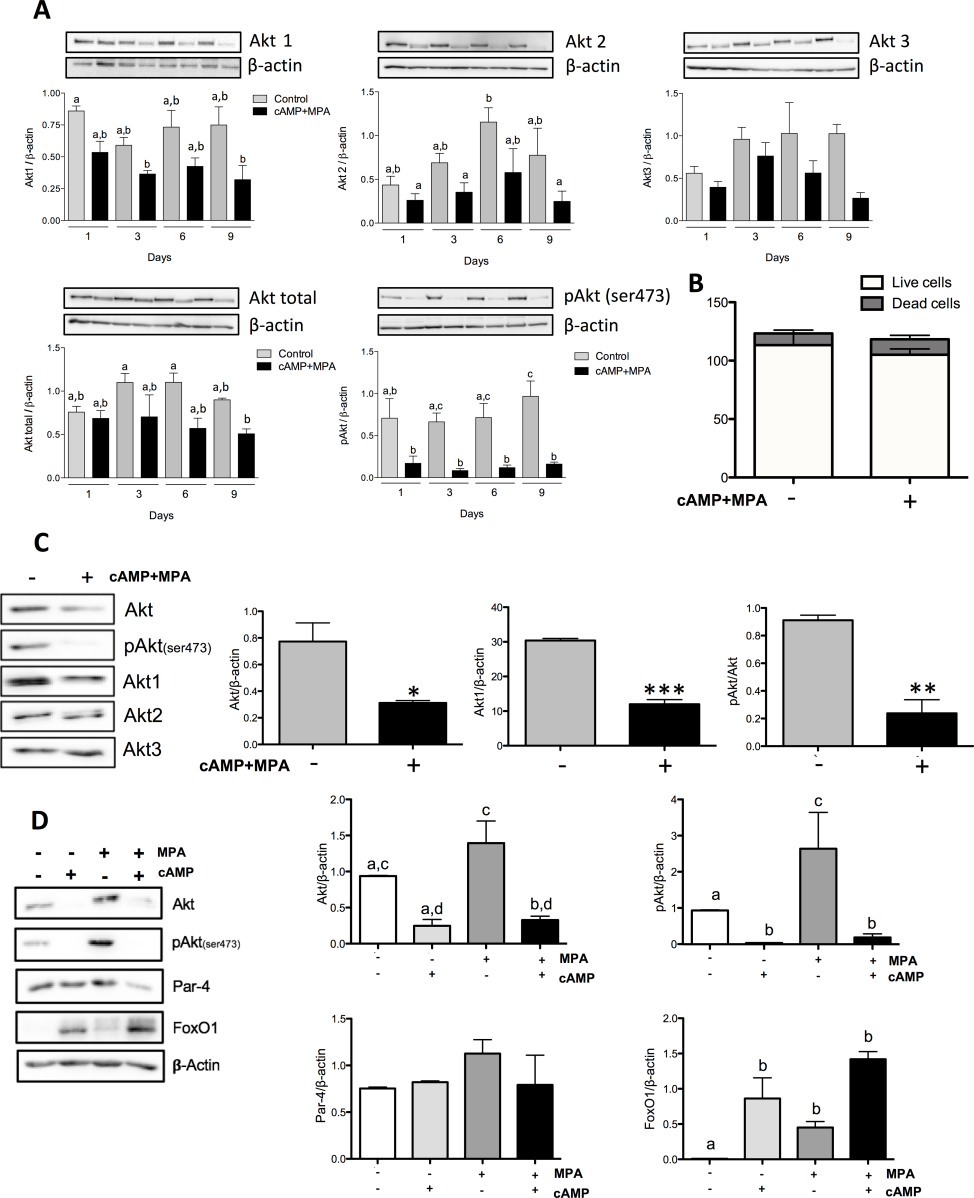
Expression of Akt isoform, total Akt and pAkt during induction of decidualization. Cells were incubated in the presence or absence of cAMP (0.5 mM) and MPA (10μM) for a total of nine days. Total protein and RNA were then extracted. (A) Western blot was performed to quantify the change in specific Akt isoforms levels as well as total Akt levels. pAkt(ser473) was used to assess Akt activation β-actin was used as loading control. (B) Cells were counted after three days in either control media or under cAMP (0.5 mM) and MPA (10μM) treatment to assess the effect of decidualization on proliferation. Trypan blue exclusion dye was used to assess the number of dead cells in the samples. (C) Cells were incubated in the presence of cAMP (0.5 mM) and MPA (10μM) treatment for three days; they were then counted; an equal amount of cells were lysed and subsequently loaded to perform Western blot analysis. Changes in total Akt, specific Akt isoforms as well as phosphorylated Akt (ser473) was quantified. (D) Cells were incubated in the presence of either cAMP (0.5 mM), MPA (10μM) or a combination of both. Cells were lysed after three days and total proteins were extracted. Western blot was performed to quantify the change in total Akt, pAkt(ser473), Par-4 or FoxO1; β-actin was used as loading control. All blots shown are from one representative experiment. All graphics represent Western blot densitometric analysis. All data are means ± SEM three independent experiments. Different letters represent significantly different means (p<0.05); *, p<0.05; **, p<0.01; ***, p<0.001.

**Fig 3:**
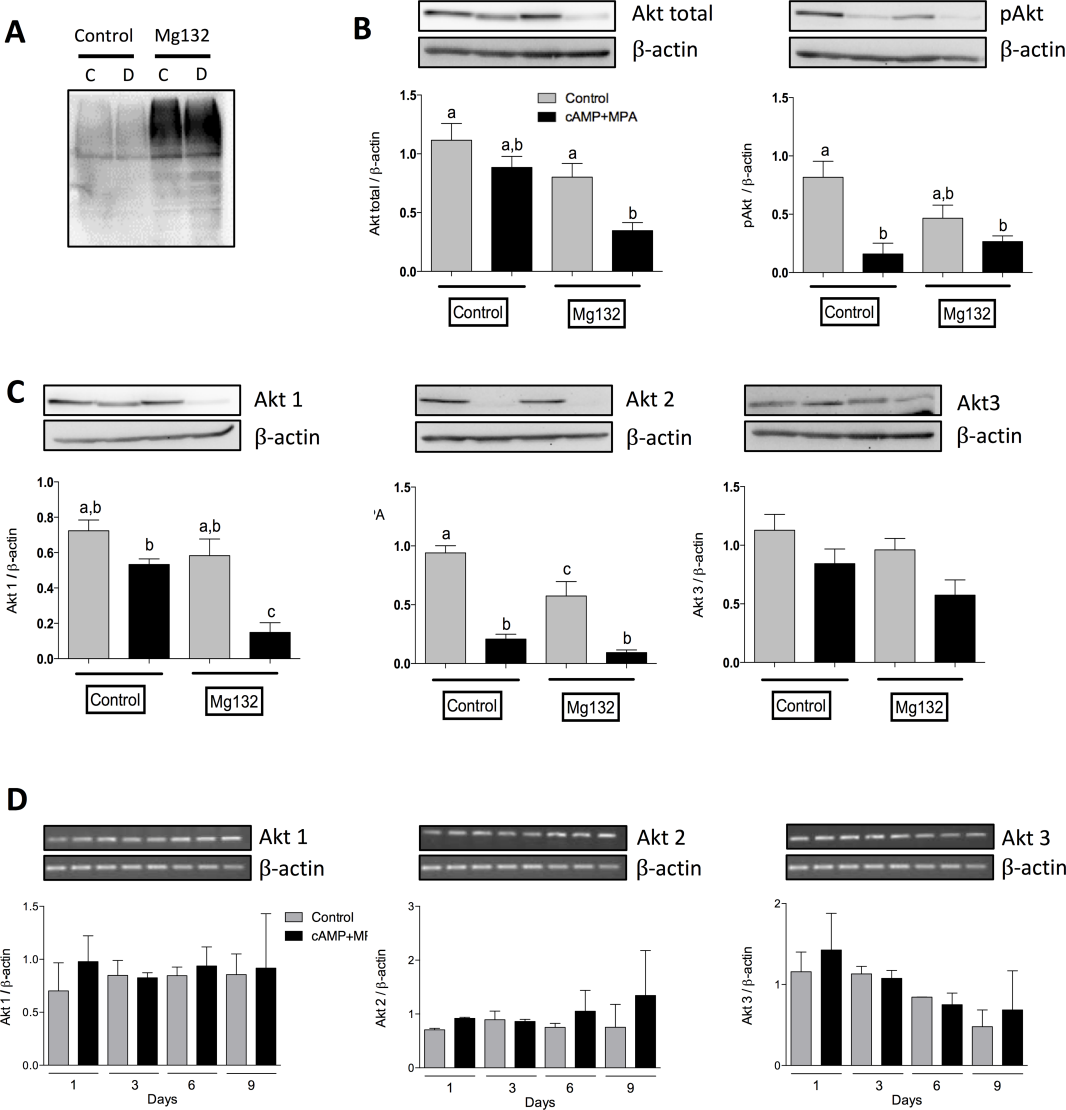
In vitro modulation of PI3K/Akt pathway with the induction of decidualization. Induction of decidualization was induced with cAMP (0.5 mM) and MPA (10μM) for 48h, then MG132 was added and incubated for another 24h. (A) Treatment using Mg132 increased total ubiquitination, both in control conditions and decidualized cells, indicating that the Mg132 was effective at inhibiting proteasomal degradation. (B) Total Akt and pAkt levels were measured by Western blot. (C) Individual Akt isoforms levels were assessed by Western blot. β-actin was used as loading control. Blots shown are from one representative experiment. Graphics represent Western blot densitometric analysis. (D) RT-PCR was performed for each Akt isoforms to evaluate change in mRNA transcription. Data represent means ± SEM for three independent experiments. β-actin was used as an internal control. Image shown are from one representative experiment. Graphics represent densitometric analysis. All data are means ± SEM three independent experiments. Different letters represent significantly different means (p<0.05).

We previously demonstrated that the PI3K/Akt pathway is crucial in the rat endometrium for cell survival [16, 17]; we thus sought to determine the expression and activity of Akt during MPA+cAMP induced decidualization. We found that protein level of each Akt isoform was decreased with the induction of decidualization, although with various levels of significance depending on the length of the treatment (Fig 2A). Additionally, we observed a decrease in total Akt and a sharp, significant decrease in phosphorylated Akt on serine 473 (pAkt (ser473)) (Fig 2A). Although some results did not attain statistical significance, a clear trend appeared in every experiment. Since decidualization is a process known to alter cell dynamics, we endeavored to evaluate the effect of decidualization on cell proliferation and viability. To this end, we proceeded to count cells after three days of cAMP+MPA treatment to evaluate the effect of decidualization on growth, and subsequent viability. We found that decidualization modestly reduced cell proliferation and slightly increased cell death, although in a non-significant manner (Fig 2B). In an effort to ascertain whether the observed protein regulations were on a cell-by-cell basis, we proceeded to decidualize HIESC cells for three days; lane loads were normalized using absolute cell counts instead of β-actin. The results showed an important, as well as significant, decrease in total Akt, pAkt (ser473) and Akt1(Fig 2C); however, as hinted by our previous experiments, Akt2 and Akt3 protein levels stayed mostly unchanged (densitometric analysis not shown). Taken together, these results suggest that, on a cell-by-cell basis, decidualization reduces Akt1 levels as well as Akt activation. In order to ascertain whether the agents used for the induction of decidualization were capable of modulating these proteins in a similar fashion, we treated HIESC with either cAMP (0.5mM), MPA (1μM) or a combination of both (Fig 2D). The obtained results suggested that cAMP was capable of significantly decreasing both Akt and pAkt. On the other hand, MPA significantly increased pAkt(473), but this upregulation was abrogated by the combination of cAMP and MPA. Interestingly, while not statistically significant, Par-4 showed a slight tendency to be downregulated by the concomitant use of cAMP and MPA and FoxO1 levels followed an upward trend. Our team recently uncovered that Par-4, a pro-apoptotic protein, acted as a cell differentiation modulator in endometrial cancer cells. After nuclear localization, Par-4 induced EMT-like phenotype and molecular alterations [28]; it is also well known that FoxO1 localization is crucial to its activity. We addressed the interrogation of whether these proteins undergo nuclear translocalization further in this manuscript. To assess the mechanism responsible for this degradation we used Mg132, a proteasome inhibitor. Although the Mg132 was effective in inhibiting the proteasome activity (Fig 3A), this inhibition did not allow the recovery of total Akt protein levels nor Akt phosphorylation following cAMP+MPA treatments (Fig 3B); further experiments also revealed that no changes could be observed in an isoform-specific manners and that Mg132 again failed to oppose the decidualization-induced loss of Akt isoforms (Fig 3C). Interestingly, RT-PCR analysis demonstrates that mRNA expression of those isoforms was not decreased (Fig 3D). These results suggest that the reduced Akt levels but unchanged transcripts levels strongly suggest that decidualization is accompanied by a degradation of Akt. Taken together, these results confirm that cAMP+MPA induced decidualization reduces Akt1 and Akt2 isoforms level and activity and that this degradation and loss of phosphorylation is not mediated by the proteasomal degradation pathway nor through transcriptional regulation of Akt isoforms expression.

### *In vitro* modulation of PI3K/Akt activity and other pathways upon induction of decidualization

**Fig 4:**
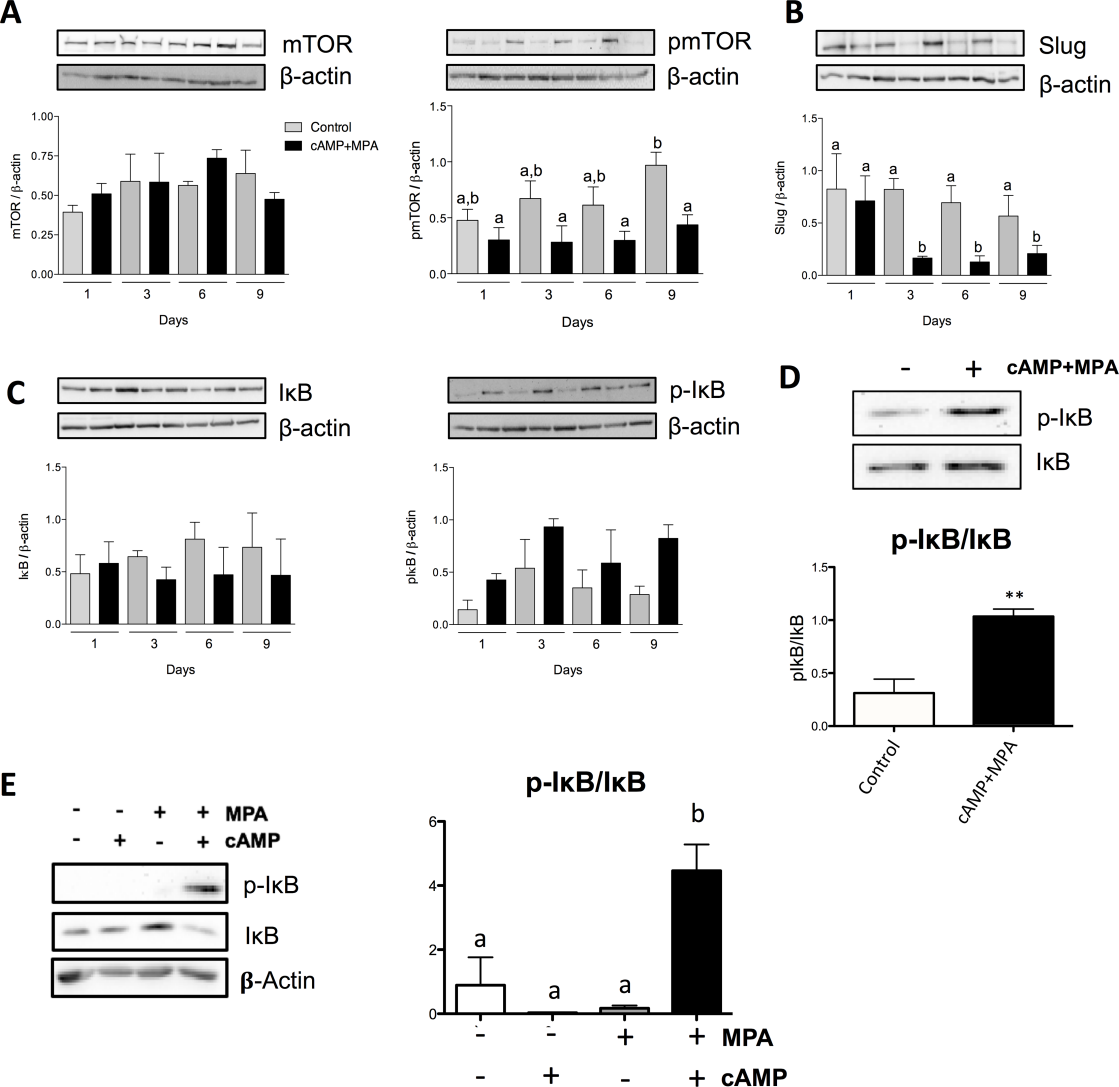
In vitro modulation of PI3K/Akt pathway and their effects with the induction of decidualization. Cells were incubated in the presence or absence of cAMP (0.5 mM) and MPA (10μM) for a total of nine days. mTOR and pmTOR protein expression (A), Slug protein expression (B) and IκB/pIκB protein expression (C) and were measured during decidualization. Total proteins were collected on different days of decidualization. β-actin was used as loading control. Blots shown are from one representative experiment. (D) Cells were harvested after three days of cAMP (0.5 mM) and MPA (10μM) treatment; they were then counted; an equal amount of cells were lysed and subsequently loaded to perform Western blot analysis. Changes in total IκB and phosphorylated IκB was quantified. (E) Cells were incubated in the presence of either cAMP (0.5 mM), MPA (10μM) or a combination of both. Cells were lysed after three days and total proteins were extracted. Western blot was performed to quantify the change in total IκB and p-IκB; β-actin was used as loading control. Presented Western blots are from one representative experiment. All graphics represent Western blot densitometric analysis. All data are means ± SEM three independent experiments. Different letters represent significantly different means (p<0.05); *, p<0.05; **, p<0.01; ***, p<0.001.

Results obtained from the previous experiments suggested that decidualization diminished Akt phosphorylation, and thus, activity. To ascertain this hypothesis, the PI3K/Akt pathway activity was evaluated by measuring the expression and activation (phosphorylation) of Akt, the main regulator of this pathway but also by measuring the phosphorylation of natural substrates of Akt; considering that Akt is known to directly activates mTOR [29], we measured the phosphorylation level of mTOR as an indicator of Akt activity. Upon cAMP+MPA treatments, we observed an increase in the levels of phosphorylated mTOR only in the control while the phosphorylation levels of mTOR remained unchanged in the treated cells (Fig 4A); while the result attained statistical significance only on day 9, a clear trend can be observed. Interestingly, some of the changes observed in the phenotype of stromal cell are reminiscent of mesenchymal to epithelial transition (MET). In order to evaluate whether classical EMT markers were involved in this change, we assessed the change in Slug, a transcription factor known to regulate epithelial to mesenchymal transition (EMT); Slug was found to be significantly decreased upon decidualization (Fig 4B). However, no significant regulations could be observed in the levels of Snail and E-Cadherin (results not shown); this suggests that, upon decidualization, HIESC cells undergo some form of, albeit incomplete, MET-like process. The obtained results, however, are insufficient to fully characterize this process.

We then further investigated the regulation of signaling cascades that might be regulated by the modulation of PI3K/Akt pathway observed during decidualization. Akt regulates the activity of the NF-κB through the activation of IKK, which phosphorylates IκB, allowing nuclear entry of NF-κB. We observed an increase in the phosphorylation of IκB (Fig 4C). This result suggests that a PI3K/Akt–independent activation of the NF-κB occurs in decidualization. Again, considering the clear trend observed in the phosphorylation of IκB, we endeavored to establish whether the magnitude of these changes would be different on a cell-by-cell basis; using absolute cell count as to normalize lane loads, we found that the increase in IκB phosphorylation, when compared to total IκB, was highly significant (Fig 4D). Finally, in order to confirm that the induction of decidualization was crucial for the phosphorylation of IκB, we again conducted experiments using cAMP and MPA both singly or in combination. The obtained results showed a significant and substantial increase in relative IκB phosphorylation solely in the context of concomitant use of both agents. These results suggest that decidualization induces the activation of the NF-κB pathway through the inactivation and subsequent degradation of its main repressor, IκB. We are also allowed to think that the combination of both agents is crucial for this pathway to be activated and is characteristic of successful decidualization.

### Effect of decidualization on Par-4 and p65 localization

**Fig 5:**
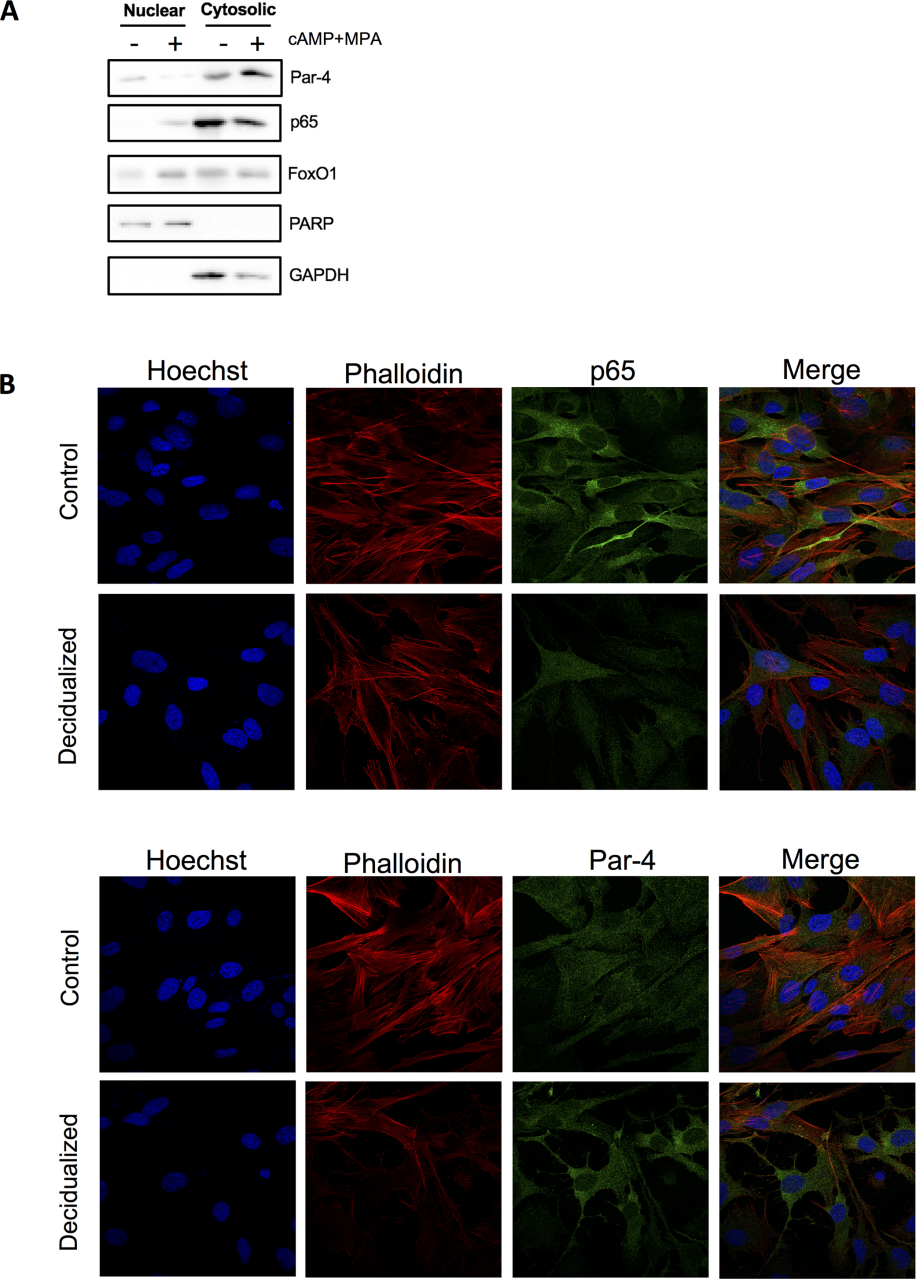
Subcellular localization of proteins following decidualization. Decidualization was induced with cAMP (0.5 mM) and MPA (10μM) for three days (A) Cells were then harvested and cytoplasmic/nuclear extraction was performed following manufacturer protocol. Western blot was performed to qualify the change in Par-4, p65 and FoxO1. PARP was used as nuclear fraction control while GAPDH was used as cytoplasmic fraction control. The presented blot is of one representative experiment. (B) HIESC were seeded in 6-wells containing coverslips and grown in presence of cAMP (0.5 mM) and MPA (10μM) for three days. p65 and Par-4 localization were then observed by confocal microscopy. Presented images are of one representative experiment.

Results obtained in the previous experiments strongly suggested that decidualization induced IκB phosphorylation, which is a marker for NF-κB activation. While not significant statistically, we observed slight Par-4 and FoxO1 modulation; as we previously demonstrated that nuclear Par-4 was a regulator of cell differentiation, we investigated its localization following decidualization. We first performed cytoplasmic/nuclear extraction to evaluate protein enrichment in both compartments in the context of day 3 decidualized HIESC cells (Fig 5A). Our results suggest that, upon decidualization, Par-4 is displaced from the nucleus while both p65 and FoxO1 are imported in the nuclear compartment. While FoxO1 displacement was well predictable, we sought to confirm our results for Par-4 and p65. To this end, we performed immunofluorescence again on day 3 decidualized cells (Fig 5B). Our results confirmed the clear displacement of Par-4 to the cytoplasm concomitant with the entry of p65 in the nucleus. Together, our results suggest that decidualization induces FoxO1 localization to the nucleus, Par-4 displacement from the nucleus to the cytoplasm and p65 entry in the nuclear compartment where its activation takes place.

### Effect of PI3K/Akt pathway inhibition on cell motility

**Fig 6:**
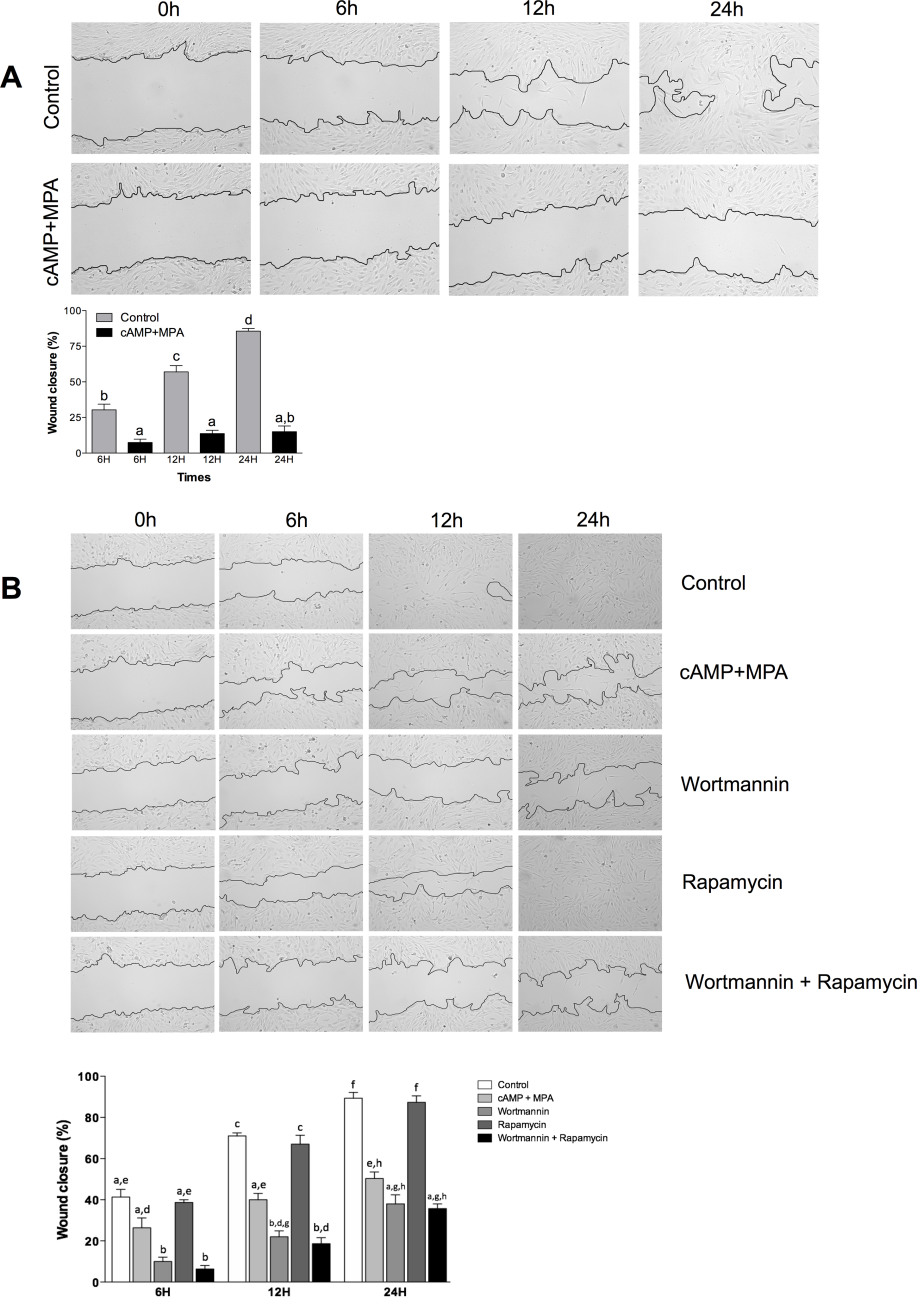
Decidualization and inhibition of PI3K/Akt pathway reduces cell motility. (A) Wound healing assays were performed using HIESC treated with either vehicle or cAMP (0.5 mM) and MPA (10μM). HIESC were allowed to grow until they reached confluence; cells monolayers were then scratched with the blunt end of a tip and images were captured at 0, 6, 12 and 24h postwounding in order to assess cell motility. Wound closure was quantified as the percentage of recovered area. **(B)** Wound healing assays were performed using HIESC treated with either vehicle or cAMP (0.5 mM) and MPA (10μM). HIESC were allowed to grow until they reached confluence; cells monolayers were then scratched with the blunt end of a tip and images were captured at 0, 6, 12 and 24h postwounding in order to assess cell motility. Treatment consisted of either vehicle (control), cAMP (0.5 mM) and MPA (10μM), 10μM Wortmannin (PI3K inhibitor) or 100 nM Rapamycin (mTOR pathway inhibitor) or combinations of these compounds. Wound closure was quantified as the percentage of recovered area. Data are means ± SEM three independent experiments. Different letters represent significantly different means (p<0.05). Images were taken using an Olympus BX60 microscope at 40x magnification.

The ability of the PI3K/Akt pathway to influence cell migration is well-documented[19]; the importance of cell motility during the embryo implantation is also recognized [30]. To evaluate the modulation of cell motility in our model, we performed a wound healing assay on decidual endometrial stromal cells. We noticed that the cAMP and MPA treatment significantly reduced cell motility, as shown by the failure of cAMP+MPA treated cell to close the created wound (Fig 6). We then sought to understand whether Akt loss, observed in the preceding experiments upon cAMP+MPA induced decidualization, was responsible for this change in cell motility. We treated the HIESC with Wortmannin, a PI3K inhibitor, and rapamycin, an mTOR inhibitor. We observed that the inhibition of the PI3K pathway by Wortmannin significantly reduced cell motility in a way very similar to the effect of cAMP+MPA induced decidualization (Fig 6). However, rapamycin failed to reduce cell motility in endometrial stromal cells. These results, coupled with the knowledge that decidualization modestly modulates cell proliferation after three days (Fig 2B), suggest that the decreased cell motility observed during decidualization seems to be associated with PI3K/Akt, independently of the mTOR pathway activity and proliferative variables.

### Effect of constitutive active Akt isoforms on the decidualization process

**Fig 7:**
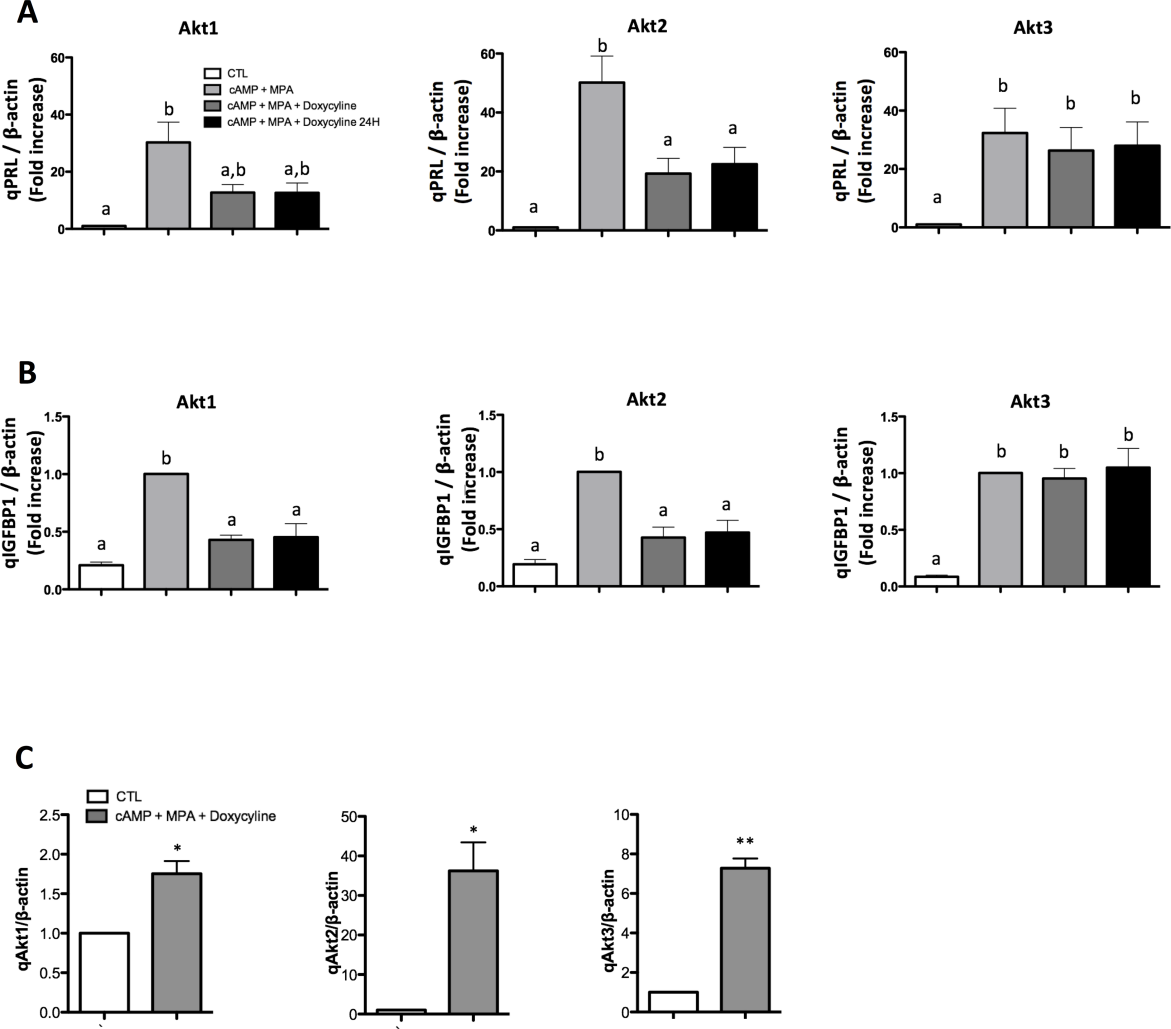
Effect of forced expression of Akt isoforms (CA-Akt) on PRL and IGFBP1 expression. mRNA expression of either PRL (A) IGFBP1 (B) were quantified following three days treatments. HIESC cells transfected with either Akt1, Akt2 or Akt3 Tet-On vectors were subjected to decidualization using cAMP (0.5 mM) and MPA (10μM). They were then either concomitantly treated with doxycycline (1μg/mL) in order to induce the expression of the constitutive Akt isoform construct (cAMP+MPA+Doxycycline) or cells were allowed to decidualize for 24h before doxycycline was added (cAMP+MPA+Doxycycline 24H). Cells were lysed after three days and qRT-PCR analyses were performed to quantify PRL or IGBP1 expression. β-actin mRNA expression was used as control for qPCR results. (C) Akt 1, Akt2 and Akt3 expression was quantified following three days treatments. HIESC cells transfected with either Akt1, Akt2 or Akt3 Tet-On vectors were subjected to decidualization using cAMP (0.5 mM) and MPA (10μM). They were then either concomitantly treated with doxycycline (1μg/mL) in order to induce the expression of the constitutive Akt isoform construct (cAMP+MPA+Doxycycline). Cells were lysed after three days and qRT-PCR analyses were performed to quantify Akt1, Akt2 or Akt3 expression. β-actin mRNA expression was used as control for qPCR results. Data are means ± SEM three independent experiments. Different letters represent significantly different means (p<0.05).

To investigate the effect of specific Akt isoforms on the decidualization process, we produced Tet-On vectors capable of expressing, upon doxycycline treatment, a constitutively active form of each Akt isoforms. The constitutive activation of Akt was achieved through the use of a myristoylation signal allowing forced localization of Akt to the membrane and facilitating its phosphorylation by its effectors. The experiment was conducted either treating the cells concomitantly with dox (to induce Akt expression) and cAMP+MPA (to induce decidualization) or the cells were decidualized and dox was applied 24h later. We decided to use these two treatment regimens in order to differentiate the effect on Akt on initialization and sustainment of decidualization. In both cases, the cells were lysed on day three and qRT-PCR was performed to quantify PRL (prolactin) mRNA expression, as a marker of successful decidualization; our experiments show that the overexpression of either Akt1 or Akt2 significantly decreased the expression of PRL while Akt3 displayed no such effect (Fig 7A); similar results were found when quantifying IGFBP1 mRNA expression (Fig 7B), further validating the inhibitory effect of Akt1-2 isoforms on the induction of decidualization. Finally, we confirmed the significant overexpression of specific Akt isoforms by quantifying their respective mRNA expressions following doxycyclin treatments (Fig 7C). Altogether, the obtained results clearly show that the timing of Akt expression does not influence the change in PRL expression. Finally, our results suggest that Akt1 and Akt2 oppose decidualization while Akt3 is uninvolved in this process.

## Discussion

Decidualization is an important event in the uterus to allow implantation. When endometrial stromal cells undergo decidualization, they differentiate and many signaling pathways are affected; the inducement of these changes can be noted by the secretion of decidual markers is induced and morphological changes are observed. cAMP and MPA co-treatment on endometrial stromal cells is associated with cell cycle arrest at G0/G1 phase initially and G2/M phase at later stages to permit differentiation of the treated cells [31]. This cell cycle arrest during decidualization can affect many pathways, in particular survival pathways, such as PI3K/Akt, one of the most pivotal pathways in determining cell fate. The aim of the present study was to characterize the expression of the PI3K/Akt survival pathway in the human endometrial stromal cells during decidualization *in vitro*.

Our study establishes that activity of Akt, as well as total protein levels, were reduced following induction of decidualization; these changes, interestingly, were isoform-specific. We demonstrated that this decrease in Akt isoforms protein levels did not involve ubiquitin-proteasome degradation. The identity of the degradation system responsible for this effect during decidualization remains unknown. The decreased activity of PI3K/Akt pathway during decidualization could affect downstream pathways responsible for cell differentiation. In the context of decidualization, fibroblasts undergo radical changes in order to adopt an epithelial-like phenotype; it is highly plausible that Akt regulates such changes in the context of decidualization. In a recent study, constitutively active Akt were expressed in squamous cell carcinoma lines and they underwent EMT. The induction of EMT was characterized by down-regulation of epithelial markers desmoplakin, E-cadherin, and β-catenin, and up-regulation of the mesenchymal marker vimentin. Those constitutively active Akt cells also exhibited reduced cell-cell adhesion, increased motility on fibronectin-coated surfaces, and increased invasiveness in animals [32]. A decrease in PI3K/Akt pathway will conduct to the reverse phenotype of this phenomenon called mesenchymal to epithelial transition (MET). When the cells undergo MET, they let go of their fibroblast-like properties and acquire epithelial cells characteristics. The morphological changes that we observed following induction of decidualization concord with this phenotype. Multiple studies have already characterized the molecular changes associated with MET phenotype switch in the process of decidualization[6–8]; however, in these studies, non-immortalized human cells were gathered from patients and treated using estradiol. Finally, some of the strongest evidence shown in these studies are drawn from mice cells, which could further explain the discrepancies observed with our results; while we observed a decrease in Slug protein level, which indicates that decidualization is accompanied by the induction of a MET-like process, although incomplete. Finally, we also observed Par-4 displacement from the nucleus to the cytoplasm following decidualization; we have recently published data demonstrating that Par-4 mediates TGF-β induced EMT[28]; while further investigation is necessary to fully understand the role of Par-4 in this partial MET, its change in localization could be a pivotal modulator of cellular differentiation.

The Akt protein is a crucial regulator of migration through its control of actin organization, cell-to-cell adhesion, cell motility and extracellular degradation. In particular, Akt1 is known to increase fibroblasts motility by phosphorylating Girdin, an actin-binding protein essential for the integrity of the actin cytoskeleton and cell migration [33]. Phosphorylation of Girdin by Akt controls its association with plasma membrane and facilitates the lamellipodium formation. Akt1 also enhances matrix metalloproteinase-2 (MMP2) activity in mouse mammary epithelial cells and invasion [34]. This proteinase is responsible for extracellular matrix modification and known to degrade the matrix components. Moreover, the influence of Akt isoform 1 and 3 are known to influence cell motility since a shRNA of those isoforms resulted in a decrease cell migration in trophoblast cells [30]. In this study, Akt 2 isoform did not result in increased or decreased cell motility. It is important to note that cell motility appears to be an important event in the successful implantation considering the invasive role of the trophoblastic cells. This motility is important in order for the implanting embryo to be fully encapsulated in the decidual tissue. The endometrial stromal cells can also affect this implantation process but it is not known how the cell motility is involved during decidualization. In our model, we observed both a decrease in cell motility, which leads to the idea that decidualization induces reduced levels of cell motility through that decreased levels of Akt isoforms. How this impacts the invasion process is still undetermined. Finally, mTOR is known to be reactivated upon cell starvation; the decidualization protocol requires the use of 2% dextran coated charcoal-treated FBS, which is far lower than the regular (10%) FBS concentration, known to contain estrogen analogs usually used in cell culture. These suggest that decidualization impedes the ability of the HIESC cells to activate the mTOR pathway in response to starvation stress, which could confer some level of resistance to autophagy[35].

Activation of PI3K/Akt is known to affect the synthesis of decidual markers[10]. During endometriosis where increase activity of this pathway is present, a decrease in the decidual marker (IGFBP1 and PRL) was observed [36]. An inhibition of Akt activity using LY294002, a PI3K inhibitor, resulted in an increase of IGFBP1 mRNA. Additionally, a majority of endometrial cancers present a mutation in PTEN or a decrease of its expression, causing an increased PI3K/Akt activity [37, 38]. Our results suggest that re-expression of those isoforms reduce the expression of IGFBP1 and PRL, two crucial markers of successful decidualization; we are thus allowed to think that Akt isoforms interferes with decidualization and that the negative regulation of Akt is necessary for the inducement of this phenotypical change. This decrease in Akt isoforms, and phosphorylated Akt, is concomitant with FoxO1 nuclear translocalisation; this is in line with the canonical series of event necessary for decidualization to occur. Interestingly, the loss of Akt activity and total protein levels were concurrent with the activation of the NF-κB pathway through the phosphorylation of its inhibitory subunit IκB. It is very well accepted that phosphorylation of IκB followed by nuclear entry of NF-κB is fully synonymous with the activation of the NF-κB transcription factor [39, 40]. While they all converge in the activation of IKK and the subsequent phosphorylation of IκB, a multitude of pathways are capable of activating the NF-κB system. One of the main actor of this regulation is, of course, the PI3K/Akt axis[41, 42]. However, considering the central role this pathway holds in cell fate, apoptosis regulation, differentiation and inflammation, the regulatory network of the NF-κB pathway is fittingly intricate. Kinases such as the NF-κB activating kinase (NIK), TGF-β activating kinase (TAK1) and MEKK1-2-3 can all mediate the activation of IKK [40, 43]; however, these signaling cascades are predominantly downstream of TNF-α, Toll-like receptors and interleukin signaling. In the context of our results, where whe observe the inhibition of the Akt axis simultaneously with an upregulation of NF-κB activity, it is conceivable that other signaling cascades are responsible. While Akt is a major modulator of NF-κB activation, multiple members of the MAPK family are also involved in its regulation; JNK and p38 have been thoroughly investigated and their involvement is definite[40, 43]. Considering that the Akt axis can exert an inhibitory effect on various arms of the MAPK pathways [44, 45], the cessation of pAkt signaling could plausibly allow a signaling compensation. There is also a distinct possibility that Akt exerts an inhibitory effect on NF-κB in the context of endometrial stromal cells, a paradoxical effect that has been shown in other contexts [46–48]. This hypothesis is interesting considering the tight regulation of inflammatory signaling during implantation, a process to which decidualization ultimately prepares; altogether, it is indisputable that further work is required to fully elucidate the nature of the molecular changes responsible for decidualization to occur.

The present study demonstrates that the PI3K/Akt has many effects during decidualization and that its forced expression interferes with this process. Considering the crucial role of this pathway in a plethora of both physiological and pathological mechanisms, our results suggest that the abnormal activity of the PI3K/Akt pathway could lead to unreceptive endometrium by impeding the orderly process of stromal decidualization.

## Supporting information

S1 Table Antibodies used in this study. Tables showing all the relevant information pertaining to the antibodies used in the context of (A)Western blot and (B) for immunofluorescence
(TIF)
