# Supplementary figures and images for: Regulation of the PI3K/Akt pathway during decidualization of endometrial stromal cells
Source: PLoS One. 2017 May 5;12(5):e0177387. doi: 10.1371/journal.pone.0177387 (PMC5419658; doi:10.1371/journal.pone.0177387)

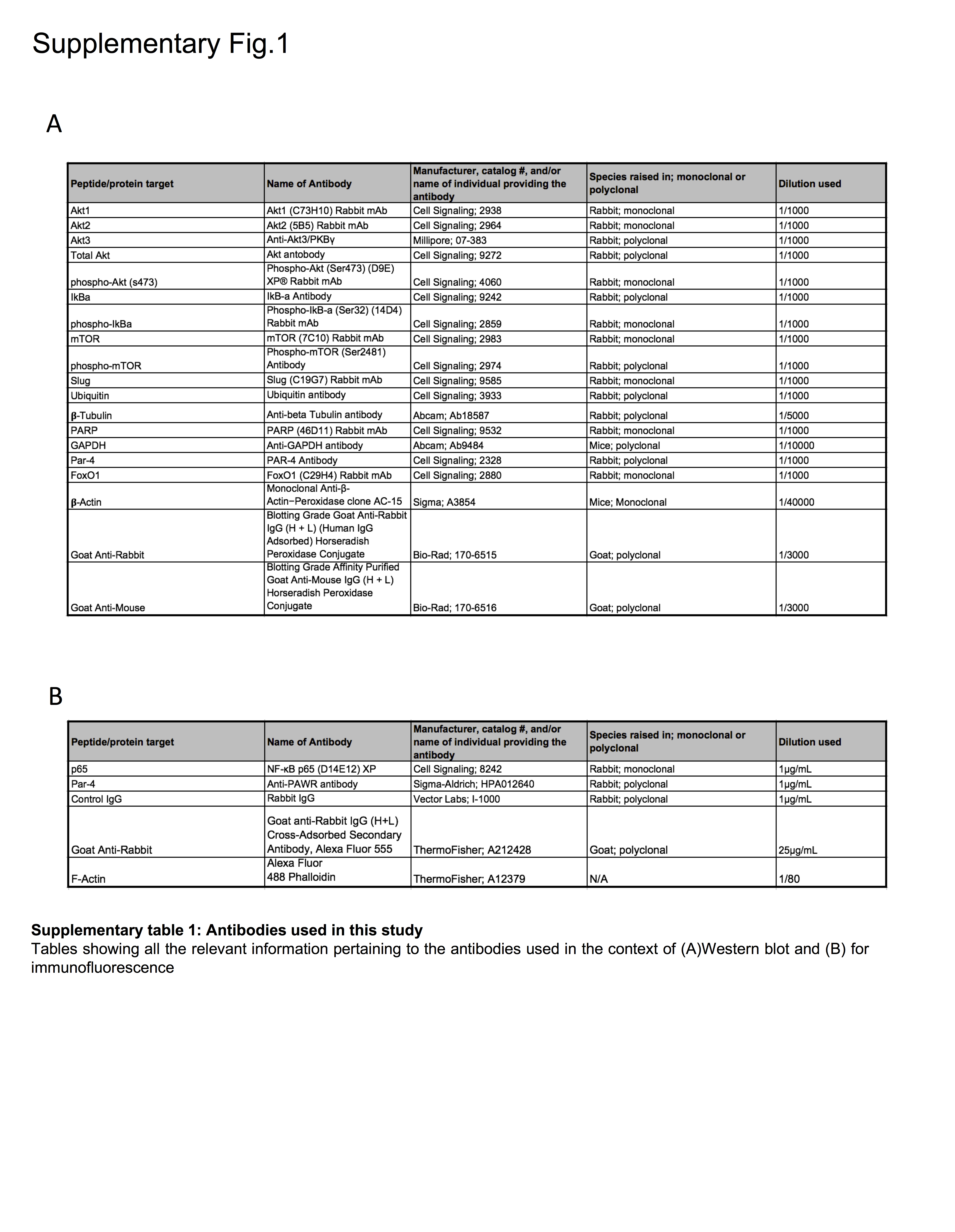

Supplement: S1 Table — Tables showing all the relevant information pertaining to the antibodies used in the context of (A)Western blot and (B) for immunofluorescence (TIF) [file pone.0177387.s001.tif]
